# 

**Published:** 1917-12-08

**Authors:** 


**Rates of Subscription:** Twelve months, 6/6; Six months, 4/-; three months, 2/-, post free to any address in the United Kingdom. Abroad. Twelve months, 8/8; Six months, 5/-; Three months, 2/6, post free. THE HOSPITAL "Index" to Volume LXII, April 1917 to September 1917, is now ready, price 2½d. per copy, post free. Address The Manager, The Hospital, "The Hospital" Building, 28/29 Southampton Street, Strand, London, W.C. 2.

### Personalia.

Dr. George Sawdon and Dr. Florence Theobalds, both of Buxton, have been elected Honorary Assistant Medical Officers to the Devonshire Hospital, Buxton, and Dr. Theobalds has also been appointed Honorary Anæsthetist.

Dr. W. H. Parkinson, who is at present serving the Warwick County Council, and who has been rejected by the military authorities, has been appointed medical officer to the Basford District Council (Notts), at a salary of £500 a year.

### HOSPITAL AND INSTITUTIONAL RESULTS.

**Glasgow Royal Infirmary.**—The infirmary received last year £39,731 in donations of £50 and upwards; these are classed as extraordinary income. The annual subscription list increased from £9,917 in 1915 to £12,222 in 1916, and the ordinary expenditure from £59,780 to £68,586. It is claimed that the accounts are now kept on the Revised Uniform System of Hospital Accounts, and there is a warning footnote to the treasurer's report that details for 1915 are consequently not comparable with 1916.

**London Fever Hospital.**—The chief feature of the medical returns for 1916 was the notable increase (from 278 to 477) in cases of German measles and the considerable falling off in scarlet fever (301 to 175). The list of governors and other benefactors runs into 175 pages. Domestic servants of governors are admitted gratuitously.

**Western Counties Institution, Starcross.**—Towards the expenditure of this institution for mental defectives, which was £16,627 in 1916, £11,626 was paid for the maintenance of patients. Subscriptions, donations, and legacies amounted to £125.

**Hornsey Cottage Hospital.**—The report for 1916 contains only bare essentials. The hospital has been open for seven years, and during that time 1,826 patients were admitted; 631 of these were received free of charge, 815 paid 5s. a week or under, and the remainder varying weekly sums up to £3 3s. The average weekly cost last year was £2 2s. 6d.

**Royal National Hospital or Consumption, Ventnor.**—The medical report for the three years 1912-1915 states that of the 2,097 cases treated, 727, or 34.7 per cent., had a family history of tuberculosis.

### Forthcoming Meetings.

League of Mercy.—The Annual Meeting of Presidents and the presentation of the Order of Mercy by H.R.H. Princess Alice, Countess of Athlone, will take place at 3 p.m. on Friday, December 21, at St. James's Palace, the Right Hon. the Viscount Farquhar, G.C.V.O., in the chair.

Royal British Nurses' Association.—Special General Meeting, 11 Chandos Street, Cavendish Square, W. 1, Wednesday, December 12, 1917, at 2.30 p.m.

### Gifts and Bequests.

Mr. William Gask, of Skirbeck, Boston, left £100 to the Boston Hospital.

The Chelsea Hospital for Women has received a grant of £40 from the Alexandra Day Fund.

By way of celebrating his eightieth birthday, Mr. Joseph Smith, a Grimsby tradesman, sent a cheque for £100 to the Grimsby Hospital.

£250 each has been left to Charing Cross Hospital, the Royal Free Hospital, and Westminster Hospital by Mr. J. H. Welby, of Sussex Place, Regent's Park, N.W.

Under the will of Mr. Henry Douglas Rhodes, 19 Buckingham Gate, S.W., chairman of the Atlas Trust, Ltd., the following bequests are to be paid on the death of his sister: £500 each to the British Home and Hospital for Incurables, Cancer Hospital, King Edward Hospital Fund, and Royal Free Hospital.

